# Quasi-Static Magnetic Field Shielding Using Longitudinal Mu-Near-Zero Metamaterials

**DOI:** 10.1038/srep12764

**Published:** 2015-08-03

**Authors:** Guy Lipworth, Joshua Ensworth, Kushal Seetharam, Jae Seung Lee, Paul Schmalenberg, Tsuyoshi Nomura, Matthew S. Reynolds, David R. Smith, Yaroslav Urzhumov

**Affiliations:** 1 Duke University, Department of Electrical and Computer Engineering, 130 Hudson Hall, Durham, North Carolina, 27708 USA; 2 University of Washington, Department of Electrical Engineering, Seattle WA 98195; 3 Toyota Research Institute of North America, Ann Arbor, Michigan, 48105 USA; 4 Toyota Central R&D Labs, Inc., Aichi, Japan

## Abstract

The control of quasi-static magnetic fields is of considerable interest in applications including the reduction of electromagnetic interference (EMI), wireless power transfer (WPT), and magnetic resonance imaging (MRI). The shielding of static or quasi-static magnetic fields is typically accomplished through the use of inherently magnetic materials with large magnetic permeability, such as ferrites, used sometimes in combination with metallic sheets and/or active field cancellation. Ferrite materials, however, can be expensive, heavy and brittle. Inspired by recent demonstrations of epsilon-, mu- and index-near-zero metamaterials, here we show how a longitudinal mu-near-zero (LMNZ) layer can serve as a strong frequency-selective reflector of magnetic fields when operating in the near-field region of dipole-like sources. Experimental measurements with a fabricated LMNZ sheet constructed from an artificial magnetic conductor – formed from non-magnetic, conducting, metamaterial elements – confirm that the artificial structure provides significantly improved shielding as compared with a commercially available ferrite of the same size. Furthermore, we design a structure to shield simultaneously at the fundamental and first harmonic frequencies. Such frequency-selective behavior can be potentially useful for shielding electromagnetic sources that may also generate higher order harmonics, while leaving the transmission of other frequencies unaffected.

A particular example in which magnetic shielding can be of vital importance is near-field wireless power transfer (WPT) systems, which have experienced a recent surge in interest with the rising popularity of mobile electronic devices. WPT schemes are also being considered to power or charge larger scale electrical devices, such as electric vehicles. Common WPT schemes involve transmitter (Tx) and receiver (Rx) coils that are magneto-inductively coupled together. Power transfer is accomplished when the magnetic flux diverging from the Tx coil is captured by the Rx coil. Because the magnetic flux diverges rapidly away from the generating coil, two challenges arise. First, the WPT efficiency is limited. At practical transfer distances *d* between Rx and Tx coils, the transfer efficiency falls off as 1/*d*^6^ due to the dipole-dipole coupling between the coils. The second challenge is managing the stray fringing of the magnetic fields that can adversely affect electronic and electrical devices as well as potentially the human body; magnetic fields must therefore be suppressed if they exceed permissible exposure levels^1^,^2^. Research efforts on WPT systems have largely focused on improving power transfer efficiency by enhancing the coupling between Tx and Rx coils. Kurs *et al.* recently demonstrated improved coupling using self-resonant coils in a four-coil WPT system^3^. A metamaterial approach to improving transfer efficiency was introduced by Wang *et al.*^4^,^5^, who suggested a negative refractive index “superlens”^6^ would help refocus the magnetic flux diverging from the Tx coil. Negative index and negative permeability materials have the unique capability to manipulate quasi-static, magnetic near-fields, and have been used in applications such as magnetic resonance imaging to guide and focus low frequency magnetic fields^7^,^8^. Subsequent analysis and experiment have also confirmed the efficacy of the superlens in enhancing coupling^9^,^10^,^11^,^12^, underscoring the potentially beneficial role of metamaterials as components in WPT systems.

Near-field focusing allows some control over the locations where magnetic flux is to be concentrated. Even more basic—yet potentially useful—functions for metamaterials are magnetic shielding and reflecting, which can also address the challenges that arise in EMI and WPT applications. Inductive WPT systems can shield magnetic fields by relying on metallic sheets and/or active field cancellation methods working together with ferrites^13^. However, when static magnetic fields need to be excluded from some region only inherently magnetic materials such as ferromagnetics and ferrites, or superconductors, can be used; that is because static magnetic field shielding is a phenomenon that requires self-sustained, non-dissipating electric currents or magnetic moments, which are only possible through quantum-mechanical effects. For low frequency magnetic fields, however, artificial magnetic media based on classical electron conductors can present an alternative medium that may offer advantages in certain scenarios. Epsilon-, index-, and mu-near-zero (MNZ) metamaterials have drawn interest recently due to their unique properties^14^,^15^,^16^; MNZ layers were discussed as means to construct perfect absorbers^17^,^18^ and can potentially shield from stray fringing fields. However, a shield acting as a reflector – rather than an absorber – can improve efficiencies of WPT systems^19^. In the following discussion we show how a LMNZ metamaterial can behave as such a magnetic field reflector.

## Results

### Shielding abilities of a longitudinal mu-near-zero layer

To understand the potential opportunities of a metamaterial shield, we first consider the Fresnel transmission and reflection formulas for a plane wave of the form 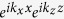 incident on a planar slab of anisotropic magnetic material, where 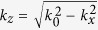 is the longitudinal wave-number (normal to the slab), and *k*_*x*_ is parallel to the slab (a derivation of these formulas can be found in the supplementary information):


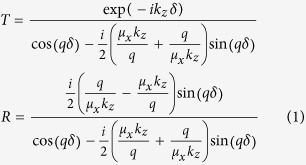


Here, 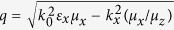 is the 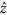-component of the wave vector within the slab medium, *δ* is the slab’s thickness, and we only consider the transmission- and reflection-coefficient for transverse electric (TE) (s-polarized) waves.

Considering Eq.(1), we see there are several possibilities for reducing the transmitted fields. For example, if either 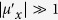 or 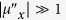, the denominator of *T* becomes large and the slab thus prevents transmission of the field (where we adopted the notation *μ*′ = Re(*μ*) and *μ*″ = Im(*μ*)). Large magnetic permeability values (up to ~1000) are available in ferrites and other soft magnetic materials, but less so in electron-conductor based metamaterials whose relative permeability tends to be limited to about 100, if realistic ohmic losses in conductors are assumed.

Fortunately, a second condition for reduced transmission occurs when the permeability takes values near zero. In fact, for TE polarization, we find it is necessary that only 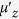 (but not 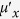) be close to zero, so that we continue our analysis assuming 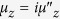 and *μ*_*x*_ = *ε*_*x*_ = 1, and refer to such a layer as longitudinal mu-near-zero (LMNZ) henceforth. In this case, 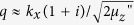, and we find that


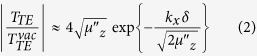


where 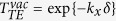 is the transmission coefficient in vacuum (in the absence of the metamaterial slab).

**Figure 1:**
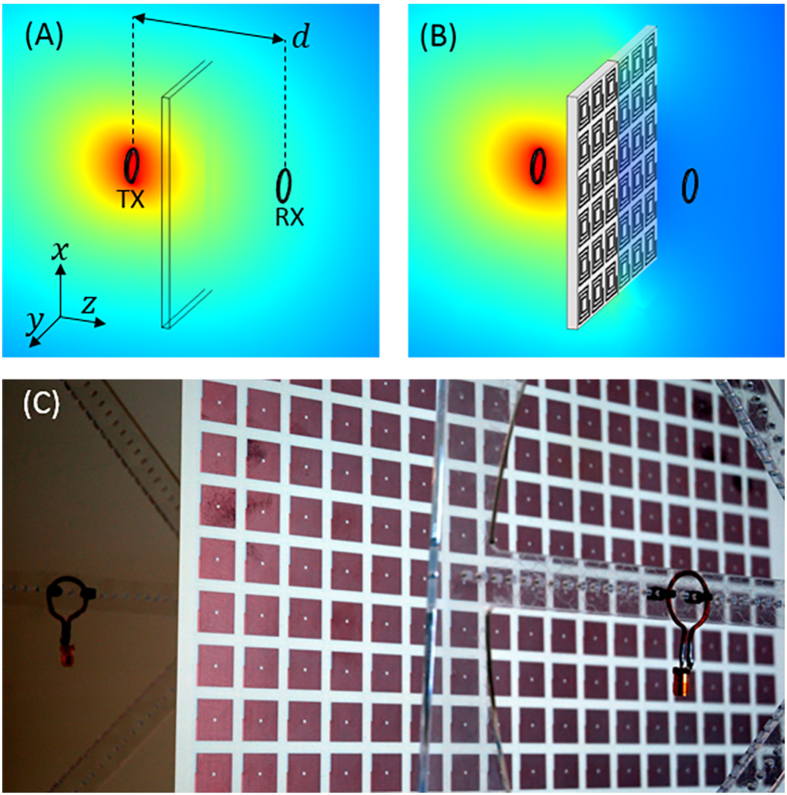
Experimental Setup. (**A**) A pair of small, non-resonant coils are separated by a distance. (**B**) When the LMNZ layer is positioned between the coils, the Rx fields are significantly attenuated (|***H***| shown in dB scale). (**C**) The experimental setup itself, showing the LMNZ sheet and two coils. Attenuation is computed from S-parameter measurements obtained with a VNA, and measurements are repeated with equally-large sheets of copper as well as the commercially available flexible ferrite FAM3 (not shown). All dimensions are specified in the text.

Eq. (2) shows that although plane waves at or near direct incidence are not reflected, the LMNZ slab will block waves at oblique angles, which contain significant portions of the energy transferred in WPT systems utilizing dipole-like coils such as the one shown in Fig. 1. In fact, from Eq. (1) it is possible to estimate those plane wave components having negligible transmission whenever the incidence angle exceeds the minimum angle given by


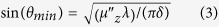


The performance of the metamaterial shield, then, is roughly determined by its thickness relative to the wavelength of operation, and the imaginary part of *μ*_*z*_.

### Implementation with a Lorentzian metamaterial layer

**Figure 2:**
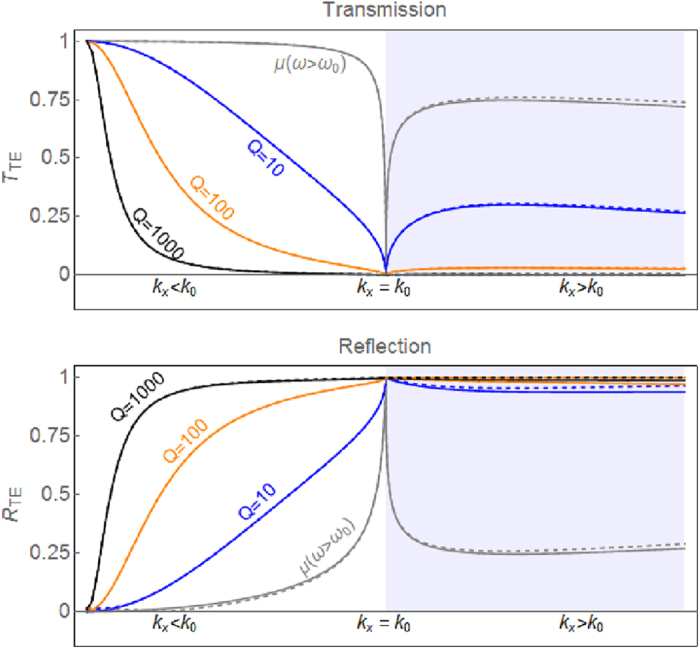
T and R of a LMNZ layer. The TE transmission (top) and reflection (bottom) coefficients (Eq. 1) are plotted as a function of ***k***_***x***_ for a LMNZ layer (solid lines) and an isotropic layer with ***μ***_***x***_ = ***μ***_***z***_ (dashed lines). At frequencies away from the ***ω*** = ***ω***_0_ point, transmission is high (gray curve). When 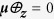, transmission is significantly reduced (and reflection enhanced), with larger attenuation for larger Q values. The LMNZ layer reflects almost as well as the isotropic layer. The layer thickness here is assumed to be *λ*/250, and the shaded region corresponds to ***k***_***x***_ > ***k***_0_, where the angle of incidence is imaginary.

An artificial magnetic metamaterial comprises split ring or spiral resonators has the generic, Lorentzian-like permeability of the form^20^


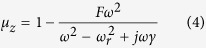


where *ω*_*r*_ is the resonance frequency, *F* is a geometric parameter smaller than unity relating to the filling fraction, and *γ* = *ω*_*r*_/(2*Q*) is a damping factor defined in terms of the resonator’s Q-factor. Using the dispersive form of *μ*_*z*_, we can quickly assess the expected shielding performance of the LMNZ layer as a function of *ω*, Q, and layer thickness. An illustrative example is shown in Fig. 2, in which a hypothetical LMNZ metamaterial slab of thickness *δ* = *λ*_0_/250 is assumed. We compute T and R at *ω*_0_, the frequency at which 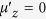 (approximated as 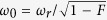) and plot T and R as a function of *k*_*x*_. While the most direct plane wave components are not reflected, within the reactive near-field zone of a dipole-like source (such as a coil), a significant amount of energy is contained in components with larger *k*_*x*_ values – including Fourier components with wavenumbers *k*_*x*_ ≫ *k*_0_ – such that the layer can be expected to act as a strong reflector. Furthermore, calculations of T and R for an isotropic slab with *μ*_*x*_ = *μ*_*z*_ (dashed lines) support our analysis that only 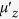 needs to be close to zero for effective shielding. Additional analysis of the LMNZ layer’s shielding abilities as a function of *μ*_*x*_ and *μ*_*z*_ is found in the supplementary material.

### Attenuation studies with non-resonant coils

To experimentally verify the attenuation capabilities of a LMNZ metamaterial we designed a metamaterial unit cell to exhibit 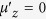 at 13.56 MHz and used standard PCB fabrication techniques to tile the unit cells and produce a LMNZ sheet 40 cm × 40 cm in size. The sheet was positioned between a pair of Tx/Rx coils separated by a distance *d* and aligned coaxially, as illustrated in Fig. 1. We formed a pair of non-resonant coils by winding a copper wire with cross-sectional diameter of 1.6 mm into a loop of radius 1 cm, connected the coils to ports 1 and 2 of an Agilent™ vector network analyzer (VNA), and recorded the S-parameters with and without the LMNZ sheet. To remove the losses associated with the mismatch between the VNA and coils when calculating the transmission coefficient, we used the measured S-parameters to compute the Max Transducer Gain^21^.

**Figure 3:**
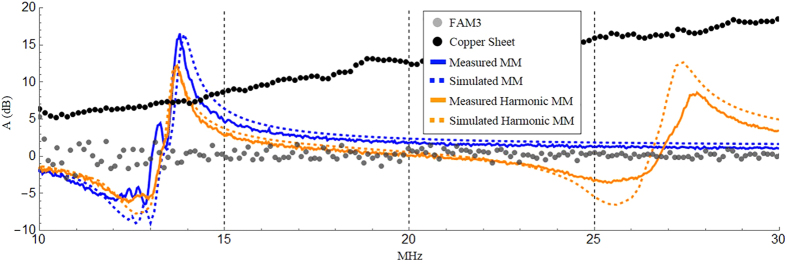
Experimental Results. Attenuation factor (dB scale) vs frequency of the measured LMNZ metamaterial (solid blue line), simulated LMNZ metamaterial (dashed blue line), measured harmonic LMNZ metamaterial (solid orange line), simulated harmonic LMNZ metamaterial (dashed orange line), measured FAM3 flexible ferrite sheet (gray disks), and measured copper sheet (black disks). The coil-to-coil distance in all cases was 11 cm.

To simulate the metamaterial’s attenuation capabilities we utilize a 2D rotationally symmetric COMSOL geometry which approximates the finite-sized LMNZ sheet as a disc of equal diameter, and use the retrieved permeability as the slab’s *μ*_*z*_. We simulate the Tx coil as a point current source a distance *r* = 1 cm from the axis of symmetry, and measure the received fields at a point on the axis of symmetry across from the slab a distance *d* away (an illustration of this geometry and more details are found in the Methods section). We define the attenuation A (in dB scale) across the slab as


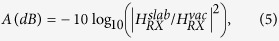


and plot the simulated and measured LMNZ attenuation factors in Fig. 3 for a coil-to-coil separation of 11 cm. We then repeated the attenuation simulation and measurement once more with a harmonic LMNZ sheet designed to exhibit shielding both at the operation frequency and at its first harmonic, and plotted the results in the same figure.

To compare our LMNZ’s shielding abilities with commercially available solutions we repeated the measurements with a sheet of FAM3 (a flexible absorbent material obtained from Digi-Key for the suppression of EMI, resonance, and coupling) as well as a sheet of Rogers 3003 circuit board, clad with 0.5oz copper (17 um thick) on both of its sides. The dimensions of both sheets (40 cm × 40 cm and 10 mil thick) were identical to our LMNZ layer, and their measured attenuation factors are plotted in Fig. 3 for the same coil-to-coil separation.

## Discussion

The agreement between the simulated and measured metamaterial behavior is excellent across the measured spectrum, for both the single-frequency and harmonic LMNZ layers. Attenuation with the LMNZ metamaterial at the 13.56 MHz operation frequency outperforms the copper and ferrite sheets by about 7 dB and 15 dB, respectively; the latter is outperformed by 5 dB–15 dB across a bandwidth of several MHz. We note that while our metamaterial is formed from 1oz copper (35 um thick) and the copper sheet is clad with 0.5oz copper (17 um thick), the total amount of copper found in both is close to identical (about half the copper is etched away in the process of fabricating the metamaterial elements). In addition, compared to conventional ferrite or metallic shields that block frequencies indiscriminately, the LMNZ layer’s frequency-selective behavior can be particularly useful in applications requiring the shielding of certain frequencies but not others. Consider WPT applications involving a communication link, for example for negotiating power delivery across the link or as a safety interlock. While a simple wideband ferrite or metallic shield would also block the communication signal, a LMNZ shield can be designed to selectively block the WPT delivery frequency with minimal effect on the communication signal. Furthermore, the LMNZ is lighter in weight than FAM3: we observe that while the slabs’ dimensions are identical, the metamaterial weighs half as much as the ferrite sheet (about 120 and 240 grams, respectively). We note that the dip in attenuation near 12.5 MHz corresponds to enhanced transmission resulting from the *μ*′ ≈ −1 superlens effect^12^. With the harmonic LMNZ layer we observe peak attenuations of 12dB and 8dB at 13.67 MHz and 27.6 MHz, respectively – within excellent range of our target frequencies.

To summarize, we have shown that a LMNZ layer – an anisotropic metamaterial slab with effective permeability *μ*_*z*_ ≈ 0 (where 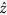 is normal to the slab) – is capable of reflecting magnetic fields with wave vector components parallel to the slab. We expect such a metamaterial to be particularly useful as a magnetic shield in WPT applications relying on dipole-like magnetic coil antennas; such systems operate within the reactive near-field zone of their source where much of the transmitted field energy is contained in fields with oblique wave vector components. Measurements of the LMNZ layer shielding performance (and of copper and FAM3, a commercially available flexible ferrite material) utilized two small non-resonant magnetic coils; experimental results indicate that the metamaterial, while much lighter than an equally large sheet of FAM3, outperforms the commercial ferrite by 5 dB–15 dB across a bandwidth of 1–3 Megahertz near its intended operation frequency. Furthermore, we designed, fabricated, and measured a multi-layer LMNZ metamaterial exhibiting shielding both at 13.56 MHz and its first harmonic.

## Methods

### Magnetic metamaterial design and fabrication

**Figure 4:**
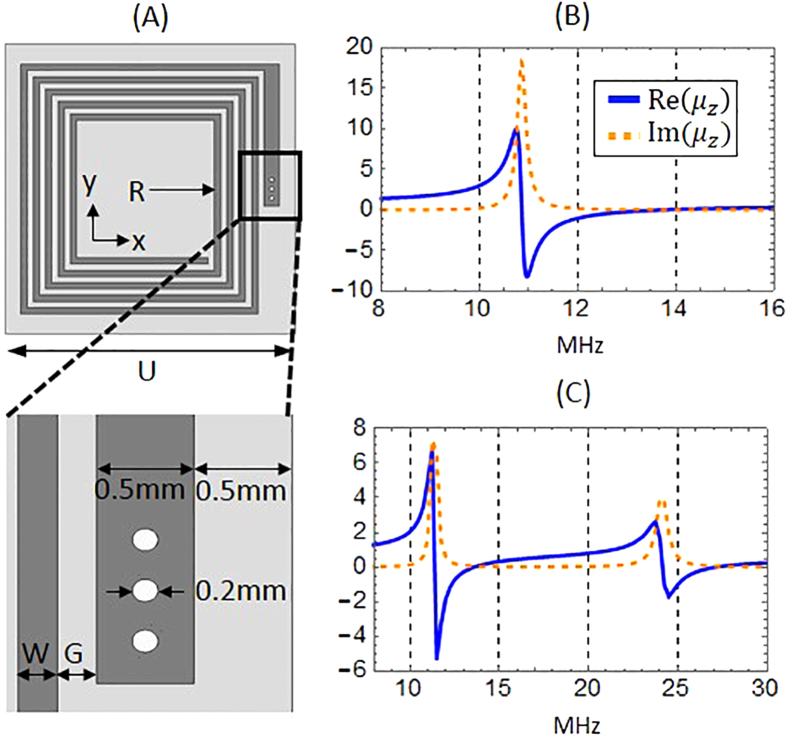
Metamaterial designs and retrieved permeability. (**A**) Simplified geometry illustrating one side of the metamaterial’s rotated-coil structure. U is the unit cell size, R is the spiral’s inner radius, W is the width of each turn and G is the gap between turns. The outer-most leg was widened to accommodate vias of radius 100 um. All dimensions are specified in the text. (**B**) The retrieved permeability of the single-layer LMNZ metamaterial exhibits 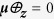 at 13.56 MHz. (**C**) The retrieved permeability of the double-layer harmonic metamaterial exhibits 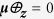 both at 13.56 MHz and its first harmonic.

It is desirable for shielding applications that the metamaterial layer be as thin as possible. To reduce the metamaterial element size relative to the wavelength (*λ*_0_ ~ 22 m), we use a variant of a spiral design^22^, in this case a double-sided rotated-coil design that is closer in size to *λ*_0_/1000, similar to that recently used in the construction of a magnetic superlens for WPT applications^12^. Using COMSOL Multiphysics, we iteratively simulate the structure and compute its permeability using the field-averaging retrieval method^12^, modifying the design until the desired properties are achieved. The final design, whose geometry and retrieved permeability are shown in Fig. 4A,B, was constructed from a 10 mil low-loss Rogers 4350 circuit board clad with 1oz copper (35 um thick) on both sides. The two arrays of spiral elements were etched on either side of the board, rotated with respect to each other and connected by vias to increase the inductance of the metamaterial element. Each spiral consisted of 17 turns, 226 um wide, with equally wide gaps between turns (W and G in Fig. 4A), except for the outer leg which was widened to 500 um in order to accommodate vias of radius 100 um. The inner radius (R) of the spirals was 2.5 mm, with the total unit cell size (U) being about 2.12 cm.

### Harmonic metamaterial design

Next we directed our efforts toward designing a double-layer LMNZ metamaterial that exhibits shielding not only at the operation frequency but also at its first harmonic. Because each layer utilizes a double-sided coil, the layers with conductive elements were separated and bonded by an insulating prepreg layer (10 mil thick) of Rogers 4450F (ε' = 3.52, δ = 0.004). It is important to note that due to the strong coupling between layers at such short distances, it is not possible to take two single-layer metamaterials sheets that exhibit attenuation bands at 13.56 MHz and 27.12 MHz when acting separately, and bond them with such a thin (10 mil) prepreg layer; the resonances experience a strong red shift when the resonators are brought that close together. Instead, the desired first and second harmonic attenuation bands must be designed with both layers already in place and separated by a pre-determined distance. The final design utilized two layers with different double-sided spirals: the first had 20 turns with R = 0.7 mm while the second had 8 turns with R = 7.5 mm (W and G in both layers were 200 um wide, and the total unit cell size U was 2.23 cm). The design’s retrieved permittivity is shown in Fig. 4C.

### Axisymmetric simulations

**Figure 5:**
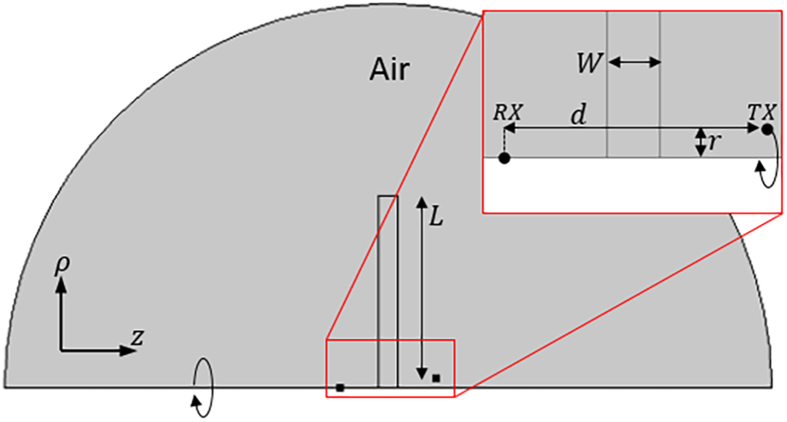
Simulated Geometry. The attenuation capabilities of the slab were simulated in COMSOL using a 2D rotationally-symmetric geometry, which approximates the finite-sized slab as a disc of equal diameter.

To simulate the experimental setup we used the 2D rotationally symmetric COMSOL geometry shown in Fig. 5. The metamaterial itself is simulated as a slab with effective permeability tensor [1, 1, *μ*_*z*_], where *μ*_*z*_ is the dispersive permeability retrieved using the Field Averaging method and plotted in Fig. 4B,C. While the experimental metamaterial sheet is rectangular in shape, due to the rotationally-symmetric nature of this simulation the slab is simulated here as a disc of equal diameter (*L* = 20 *cm*). The width of the simulated slab (*W*, set to 2 cm in simulations) is determined based on the corresponding value used during the field-averaging retrieval, and is not necessarily equal to the physical thickness of the fabricated metamaterial. We simulated the Tx coil as a point current source with fixed current I = 1 A at a radius *r* = 1 cm from the axis of symmetry, and measured the received fields *H*_*z*_ as a function of frequency at a point on the axis of rotation a distance *d* from the transmitting coil, corresponding to the location of the Rx coil center.

## Additional Information

**How to cite this article**: Lipworth, G. *et al.* Quasi-Static Magnetic Field Shielding Using Longitudinal Mu-Near-Zero Metamaterials. *Sci. Rep.* **5**, 12764; doi: 10.1038/srep12764 (2015).

## Supplementary Material

Supplementary Information

## References

[b1] ICNIRP Guidelines for limiting exposure to time-varying electric, magnetic, and electromagnetic fields (up to 300 GHz). Health Phys 74, 494–522 (1998). 9525427

[b2] ICNIRP Report, Guidelines for limiting exposure to time-varying electric and magnetic fields (1 Hz to 100 kHz). Health Phys. 99, 818–836 (2010). 21068601 10.1097/HP.0b013e3181f06c86

[b3] Kurs A. *et al.* Wireless power transfer via strongly coupled magnetic resonances. Science 317, 83–86 (2007). 17556549 10.1126/science.1143254

[b4] Wang B. *et al.* Wireless power transfer with metamaterials. Proceedings of the 5th IEEE European Conference on Antennas and Propagation (EUCAP), 3905–3908 (2011).

[b5] Wang B., Nishino T. & Teo K. H. Wireless power transmission efficiency enhancement with metamaterials. Proceedings of the IEEE International Conference on Wireless Information Technology and Systems (ICWITS), 28, 1–4 (2010). 10.1109/ICWITS.2010.5612284

[b6] Pendry J. B. Negative refraction makes a perfect lens. Phys. Rev. Lett. 85, 3966–3969 (2000). 11041972 10.1103/PhysRevLett.85.3966

[b7] Wiltshire M. C. K. *et al.* Microstructured magnetic materials for RF flux guides in magnetic resonance imaging. Science 291, 849–851 (2001). 11157159 10.1126/science.291.5505.849

[b8] Wiltshire M. C. K., Hajnal J. V., Pendry J. B., Edwards D. J. & Stevens C. J., Metamaterial endoscope for magnetic field transfer: near field imaging with magnetic wires. Optics Express 11, 709–715 (2003). 19461782 10.1364/oe.11.000709

[b9] Wang B. *et al.* Experiments on wireless power transfer with metamaterials. Appl. Phys. Lett. 98, 254101 (2011).

[b10] Urzhumov Y. & Smith D. R. Metamaterial-enhanced coupling between magnetic dipoles for efficient wireless power transfer. Phys. Rev. B 83, 205114 (2011).

[b11] Huang D., Urzhumov Y., Smith D. R., Teo K. H. & Zhang J. Magnetic superlens-enhanced inductive coupling for wireless power transfer. J. Appl. Phys. 111, 064902 (2012).

[b12] Lipworth G. *et al.* Magnetic metamaterial superlens for increased range wireless power transfer. Scientific Reports 4, 3642 (2014). 24407490 10.1038/srep03642 PMC3887385

[b13] Kim J. *et al.* Coil design and shielding methods for a magnetic resonant wireless power transfer system. Proc. IEEE 101, 1332–1342 (2013).

[b14] Silveirinha M. & Engheta N. Tunneling of electromagnetic energy through subwavelength channels and bends using ε-near-zero materials. Phys. Rev. Lett. 97, 157403 (2006). 17155357 10.1103/PhysRevLett.97.157403

[b15] Alù A., Silveirinha M. G., Salandrino A. & Engheta N. Epsilon-near-zero metamaterials and electromagnetic sources: Tailoring the radiation phase pattern. Phys. Rev. B, 75, 155410 (2007).

[b16] Hao J., Yan W. & Qiu M. Super-reflection and cloaking based on zero index metamaterial. Applied Physics Letters, 96, 101109 (2010).

[b17] Zhong S. & He S. Ultrathin and lightweight microwave absorbers made of mu-near-zero metamaterials. Scientific Reports, 3 (2013). 10.1038/srep02083 PMC3694291 23803861

[b18] Jin Y., Xiao S., Mortensen N. A. & He S. Arbitrarily thin metamaterial structure for perfect absorption and giant magnification. Optics express, 19, 11114–11119 (2011). 21716340 10.1364/OE.19.011114

[b19] Wu J., Wang B., Yerazunis W. S. & Teo K. H. Wireless power transfer with artificial magnetic conductors. Wireless Power Transfer (WPT), 2013 IEEE, 155–158 (2013). 10.1109/WPT.2013.6556906

[b20] Pendry J. B., Holden A. J., Robbins D. J. & Stewart W. J. Magnetism from conductors and enhanced nonlinear phenomena. Microwave Theory and Techniques, IEEE Transactions on, 47, 2075–2084 (1999).

[b21] Pozar D. M. in Microwave Engineering 4th edn, 571–572 (John Wiley & Sons, 2012).

[b22] Chen W. C., Bingham C. M., Mak K. M., Caira N. W. & Padilla W. J. Extremely subwavelength planar magnetic metamaterials. Phys. Rev. B 85, 201104 (2012).

