# Supplementary material for: Quasi-Static Magnetic Field Shielding Using Longitudinal Mu-Near-Zero Metamaterials
Source: Sci Rep. 2015 Aug 3;5:12764. doi: 10.1038/srep12764 (PMC4522651; doi:10.1038/srep12764)
Supplement: Supplementary Information [file srep12764-s1.pdf]

Quasi-Static Magnetic Field Shielding Using Longitudinal Mu-Near-Zero Metamaterials

Guy Lipworth[1], Joshua Ensworth[2], Kushal Seetharam[1], Jae Seung Lee[3], Paul Schmalenberg[3], Tsuyoshi Nomura[4], Matthew S. Reynolds[2], David R. Smith[1,*], Yaroslav Urzhumov[1]

[1]Duke University, Department of Electrical and Computer Engineering, 130 Hudson Hall, Durham, North Carolina, 27708 USA;
[2]University of Washington, Department of Electrical Engineering, Seattle WA 98195;
[3]Toyota Research Institute of North America, Ann Arbor, Michigan, 48105 USA;
[4]Toyota Central R&D Labs, Inc., Aichi, Japan;
*e-mail: drsmith@duke.edu

Supplementary Information

As explained in the main text, one motivation for shielding using a longitudinal-mu-near-zero (LMNZ) metamaterial is the difficulty associated with designing high-μ metamaterials. In addition, a second problem plagues solutions relying on high-μ shielding: the permeability of ferrites typically begins to fall rapidly at frequencies above 30 MHz, which leads to a "frequency gap" between high-permeability and high-conductivity based solutions for quasi-static magnetic field screening. This gap is particularly pronounced for TE-polarized magnetic field sources, for which the magnetic field transmission coefficient is almost entirely independent of the complex dielectric constant (in the quasi-static limit), and therefore screening solutions based on effective electrical conductivity are very inefficient.

Transmission and Reflection in Anisotropic Medium

To simplify the analysis leading to eq.1 in the main text, we restrict our attention to cases where the incoming wave is either TE or TM. Furthermore, we'll assume the only components of ε and μ that matter are the transverse component of ε (along $\hat{y}$) and the longitudinal component of μ (along $\hat{z}$). In general,

$$\begin{aligned} \nabla \times \mathbf{H} &= -i\omega\bar{\bar{\varepsilon}}\mathbf{E} \\ \nabla \times \mathbf{E} &= +i\omega\bar{\bar{\mu}}\mathbf{H} \end{aligned} \qquad (1.1)$$

Considering the geometry shown below for S-polarization (TE), we observe the only electric field component is E_y, and the only magnetic field components are H_x and H_z.

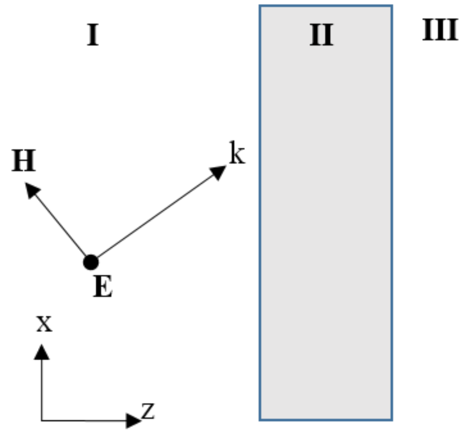

Figure S1 S-polarized (TE) wave incident on a slab

Maxwell's equations for this scenario can be written as:

$$\frac{\partial H_z}{\partial x} - \frac{\partial H_x}{\partial z} = -i\omega\varepsilon_y E_y$$
$$H_x = \frac{1}{i\omega\mu_x}\frac{\partial E_y}{\partial_z} \quad (1.2)$$
$$H_z = \frac{1}{i\omega\mu_z}\frac{\partial E_y}{\partial_x}$$

and we combine these to yield:

$$\frac{1}{\mu_z}\frac{\partial^2 E_y}{\partial x^2} + \frac{1}{\mu_x}\frac{\partial^2 E_y}{\partial z^2} = -\omega^2\varepsilon_y E_y \qquad (1.3)$$

We note only the components ε_y, μ_x, and μ_z enter the equation, as is expected for an S-polarized wave. For soluaions of the form $E_y = \exp(iq_x x + iq_z z)$ we obtain the dispersion relation $\frac{q_x^2}{\mu_z} + \frac{q_z^2}{\mu_x} = \omega^2\varepsilon_y$. In free-space, of course, the wave solution has the corresponding dispersion relation $k_x^2 + k_z^2 = \omega^2$. We now divide the space into three regions as depicted in Figure S1: free-space for $z < 0$ and $z > \delta$, and anisotropic medium for $0 < z < \delta$. Assuming the wave is incident on the slab from the left, the solutions for each region are:

$$\begin{aligned} &\mathrm{I}: \quad E_y(x,z) = e^{ik_x x}e^{ik_z z} + re^{ik_x x}e^{-ik_z z} \\ &\mathrm{II}: \; E_y(x,z) = ae^{ik_x x}e^{iq_z z} + be^{ik_x x}e^{-iq_z z} \qquad (1.4) \\ &\mathrm{III}: E_y(x,z) = te^{ik_x x}e^{iq_z z} \end{aligned}$$

where we used the continuity of transverse moment ($q_x = k_x$) and the fact there is only an outgoing wave on the right side. There are now four unknowns left to solve for (*a*,*b*,*r,* and *t*) and two boundaries at which E_y and its derivative must be continuous. Alternatively, E_y and H_x must be continuous, such that we have enough conditions to solve for *t* and *r* in terms of ε_y , μ_x , μ_z , ω , and δ . This provides the Fresnel formula for a wave at oblique angles of incidence to an anisotropic slab (and in the same manner we can compute the solutions for the TM case). To solve for *a* and *b* we complete the boundary value problem, where for the $z = \delta$ interface we have:

$$a = \frac{1}{2}\left(1 + \frac{\mu_x k_z}{q_z}\right) e^{i(k_z - q_z)\delta} t$$
$$b = \frac{1}{2}\left(1 - \frac{\mu_x k_z}{q_z}\right) e^{i(k_z + q_z)\delta} t \quad (1.5)$$

and for the $z = 0$ interface we have:

$$2 = \left(1 + \frac{q_z}{u_x k_z}\right) a + \left(1 + \frac{q_z}{u_x k_z}\right) b$$
$$2 = \left(1 - \frac{q_z}{u_x k_z}\right) a + \left(1 - \frac{q_z}{u_x k_z}\right) b \quad (1.6)$$

Combining the above yields eq(1) in the main text (where we replace q_z with q):

$$T = \frac{\exp(-ik_z\delta)}{\cos(q\delta) - \frac{i}{2}\left(\frac{\mu_x k_z}{q} + \frac{q}{\mu_x k_z}\right)\sin(q\delta)}$$
$$R = \frac{\frac{i}{2}\left(\frac{q}{\mu_x k_z} - \frac{\mu_x k_z}{q}\right)\sin(q\delta)}{\cos(q\delta) - \frac{i}{2}\left(\frac{\mu_x k_z}{q} + \frac{q}{\mu_x k_z}\right)\sin(q\delta)} \quad (1.7)$$

Additional Characteristics of the LMNZ Shielding Regime

As shown by eq.3, the performance of the metamaterial shield is roughly determined by its thickness relative to the wavelength of operation, and by μ_z''. Interestingly, the shielding effect can be obtained regardless of the sign of μ_x' . Very similar expressions can be obtained assuming, for example, that μ_x' equals -1 or any negative value not too close to zero, and regardless of the magnitude of μ_x'' . The strong anisotropy of magnetic permeability is, on the other hand, critical for the $|q| \to \infty$ shielding regime to exist, as this effect is not present when both μ_x and μ_z are small; this phenomenon is illustrated in Figure S2. The longitudinal mu-near-zero (LMNZ) regime is therefore fundamentally different from the MNZ regime studied in references [3,17]. One may notice that the LMNZ regime is a “topological transition” between the definite (all-positive or all-negative) and indefinite permeability regime, which has been shown to have some peculiar electromagnetic properties both with respect to far-field and near-field phenomena [s1].

Another notable feature of the LMNZ shielding regime is its surprisingly high bandwidth. Consider a frequency at which the real part of longitudinal permeability is detuned from zero, and the loss tangent is one: $\mu_z = \mu''(1+i)/\sqrt{2} = \mu'' e^{i\pi/4}$. At that frequency, the transmission coefficient (2) becomes

$$\left|\frac{t_{TE}}{t_{TE}^{vac}}\right| \approx 4\times 2^{1/4}\sqrt{\mu''}\exp\left\{-\frac{k_x d}{\sqrt{\mu''}}\cos\left(\frac{\pi}{8}\right)2^{1/4}\right\} \qquad (1.8)$$

The pre-exponential factor in this expression is different from the corresponding factor in Eq.2 by only $2^{1/4} \approx 1.2$, and the exponential coefficient is different only by a factor of $\cos(\pi/8)2^{1/4}/\sqrt{2} \approx 1.1$. The transmission coefficient is therefore very flat as a function of frequency near the LMNZ point.

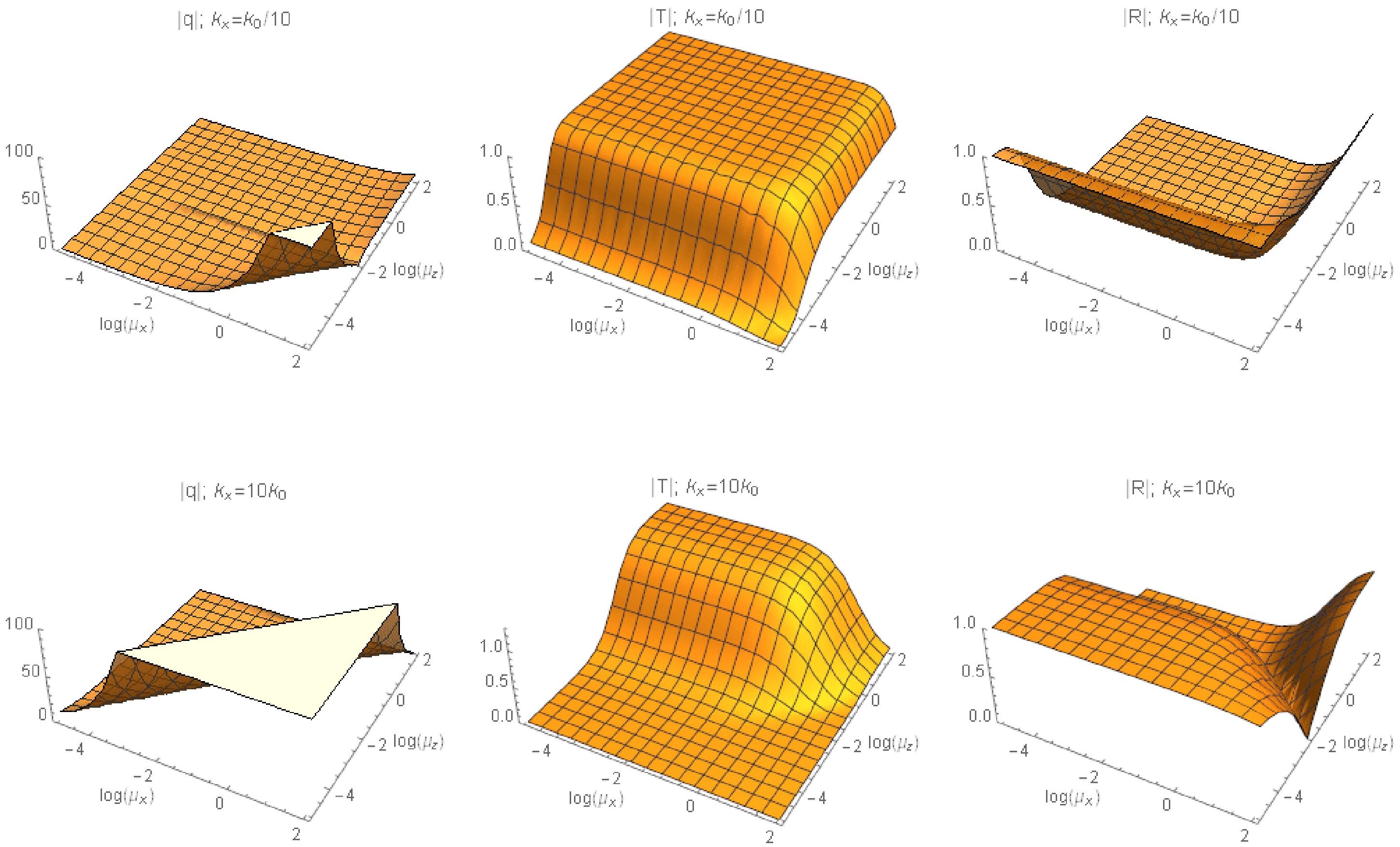

Figure S2 | $|q| \to \infty$ shielding regime vs. μ_x and μ_z. |q|, |T|, and |R| are plotted for a layer that is $\lambda/250$ thick, as a function of μ_z' and μ_x' for $k_x < k_0$ (top row) and $k_x > k_0$ (bottom row). To observe the $|q| \to \infty$ shielding regime the metamaterial layer must exhibit anisotropic permeability: only μ_z' should be near-zero (left). Transmission (center) may still decrease when μ_x' is near-zero as well, but $|T| \to 0$ is observed only in the regions where $|q| \to \infty$.

Rotationally-Symmetric Simulations of a Coil Enclosed by a Shell

To confirm the simple analytic argument, we use a 2D rotationally-symmetric COMSOL geometry to simulate a transmitting coil enclosed by an isotropic shell composed of three planar slabs, as shown in Fig. S3A. Here the axis of symmetry lies along the $\hat{z}$-axis and the transmitting coil loop is simulated as a 1A point current source a distance $r = 1\text{cm}$ from the axis of symmetry. First, we sweep the relative permeability of the slabs from low values ($\mu' = 0.01$) to high values ($\mu' = 100$). We then repeat this calculation with diagonally anisotropic slabs and sweep only the component of μ normal to each slab across the same range of values (Fig. S3B). In both cases μ'' is kept at ideal value of zero. In Fig. S3C we show the magnetic field norm, integrated along the arc portion of the external simulation boundary, for both cases. First, we observe that a shell with isotropic permeability can achieve effective shielding not only when μ' is very high, but also when μ' is near zero. Second, we note that for low values of μ', shielding with the LMNZ slabs is almost as effective as with the isotropic slabs.

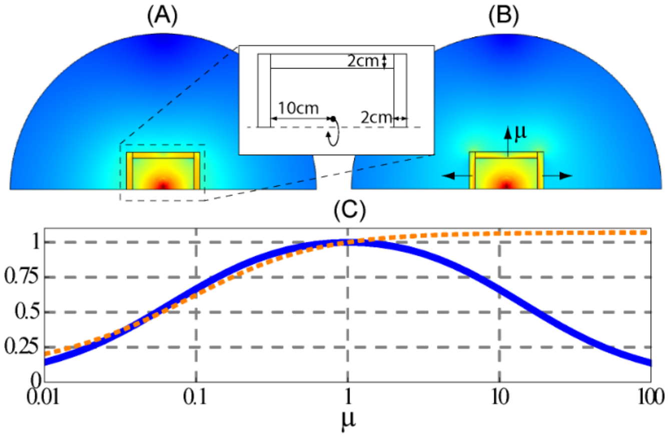

Figure S3 | Transmission through isotropic and longitudinal mu-near-zero metamaterials. A Tx coil surrounded by a shell composed of three planar slabs is simulated using COMSOL's rotationally-symmetric geometry. The magnetic fields in the domain (dB scale) are shown for isotropic μ-near-zero (A) and longitudinal μ-near-zero (B) shells. In (C) we show H_{norm} integrated along the domain's external arc as a function of the relative permeability for both the isotropic (solid blue line) and longitudinal (dotted orange line) layers. The values are normalized by the $\mu = 1$ case.

Supplementary References

1. Harish N. S. Krishnamoorthy, Zubin Jacob, Evgenii Narimanov, Ilona Kretzschmar, Vinod M. Menon, "Topological transitions in metamaterials," Science 336 no. 6078 pp. 205-209.
